# Association Between Systemic Immune‐Inflammation Index and Thyroid Function: A Cross‐Sectional Population‐Based Study

**DOI:** 10.1155/ije/7554085

**Published:** 2026-01-07

**Authors:** Jiaqi Huang, Jieqiong Song, Ming Zhong, Fei Leng

**Affiliations:** ^1^ Department of Urology, Zhongshan Hospital, Fudan University, Shanghai, China, fudan.edu.cn; ^2^ Department of Urology, Minhang Hospital, Fudan University, Shanghai, China, fudan.edu.cn; ^3^ Department of Critical Care Medicine, Zhongshan Hospital, Fudan University, Shanghai, China, fudan.edu.cn

**Keywords:** cross-sectional study, NHANES, systemic immune-inflammation index, thyroid function

## Abstract

**Background:**

The systemic immune‐inflammation index (SII), an emerging inflammatory biomarker, has been associated with various diseases, but its relationship with thyroid function remains unclear. Our study aimed to investigate the potential connections between SII and thyroid function in the U.S. population.

**Methods:**

We conducted a cross‐sectional study using the National Health and Nutrition Examination Survey (NHANES) 2007–2012 data to evaluate the association between SII and thyroid function, including free triiodothyronine (FT3), free thyroxine (FT4), total triiodothyronine (TT3), total thyroxine (TT4), and thyroid‐stimulating hormone (TSH). Furthermore, the correlation was evaluated using multiple linear regression, smooth curve fitting, and threshold effect analysis.

**Results:**

After multivariable linear regression, higher lgSII levels were independently associated with lower FT3 (*β* = −0.17, *p* < 0.0001), TT3 (*β* = −3.06, *p* = 0.0149), and TSH (*β* = −0.38, *p* = 0.0204), whereas TT4 levels were positively associated with lgSII after full adjustment (*β* = 0.27, *p* = 0.0016). Smooth curve fitting revealed an L‐shaped relationship between lgSII and FT3 and TSH. Threshold effect analysis identified an inflection point at lgSII = 2.29 (log‐likelihood ratio, *P* < 0.001).

**Conclusion:**

In U.S. adults, lgSII was negatively associated with FT3 and TSH and positively associated with TT4. These findings highlight a potential link between systemic inflammation and thyroid function, warranting further prospective studies to investigate causal relationships.

## 1. Introduction

The systemic immune‐inflammation index (SII), calculated as the product of platelet and neutrophil counts divided by lymphocyte count, was first proposed by Hu et al. in 2014 and has been since extensively investigated [1]. The SII has gained attention as a nonspecific marker that reflects both systemic inflammation and local immune responses [2, 3]. It is commonly used as a prognostic marker for tumor patients, as elevated SII levels have been associated with increased mortality risk [4, 5]. Additionally, Yang et al. demonstrated that SII had superior predictive value for major cardiovascular events in patients undergoing coronary intervention compared with traditional risk factors [6]. Over recent years, the application of SII has expanded, demonstrating its potential to predict the severity of various conditions, including hyperlipidemia [7], chronic obstructive pulmonary disease (COPD) [8], renal disorders [9, 10], autoimmune diseases [11], and other diseases [12]. In addition, the application of SII in nonthyroid inflammatory diseases has been systematically reported. For example, Okutan et al. [11] demonstrated its association with disease activity in rheumatoid arthritis, whereas Aci et al. [13] reported its predictive value in gestational diabetes mellitus. Other studies have linked SII with chronic kidney disease outcomes, COPD prognosis, and sarcopenia‐related mortality. By integrating these findings in a chronological manner, we further highlight SII as a broadly applicable biomarker, which strengthens the rationale for exploring its relationship with thyroid function.

The mammalian growth, neural differentiation, and metabolic regulation depend on the thyroid hormones (THs) [14]. Even deviations in TH levels, without necessarily indicating overt disease, can result in impaired cognitive function, increased susceptibility to cardiovascular disease [15], osteoporosis [16], and cognitive decline [17].

Preliminary evidence indicates that systemic inflammation can alter cytokine profiles, trigger oxidative stress, and impair thyroid function, ultimately disrupting the hypothalamic–pituitary–thyroid (HPT) axis [18]. In recent years, several studies have indicated that SII may have potential value in the diagnosis and prognostic assessment of thyroid cancer [19, 20]. Furthermore, Serpil Ciftel et al. demonstrated that SII serves as an indicator of the inflammatory process in subacute thyroiditis (SAT) and can be used to predict the recovery time of SAT. These findings suggest a potential association between SII and thyroid disease [21]. However, the precise relationship between SII and thyroid function remains unclear.

This study investigates the cross‐sectional association between SII and thyroid function to address the previously undescribed issues and offer more evidence. This analysis will use data from the National Health and Nutrition Examination Survey (NHANES) conducted in the United States between 2007 and 2012, representing a diverse sample of adults.

## 2. Method

### 2.1. Study Population

We analyzed NHANES data collected during six survey‐year cycles from 2007 to 2012 in this cross‐sectional study. The National Center for Health Statistics (NCHS) Ethics Review Committee approved the NHANES investigation protocol, and the original NHANES data were retrieved for this study. Written informed consent was obtained from all participants.

**Figure 1:**
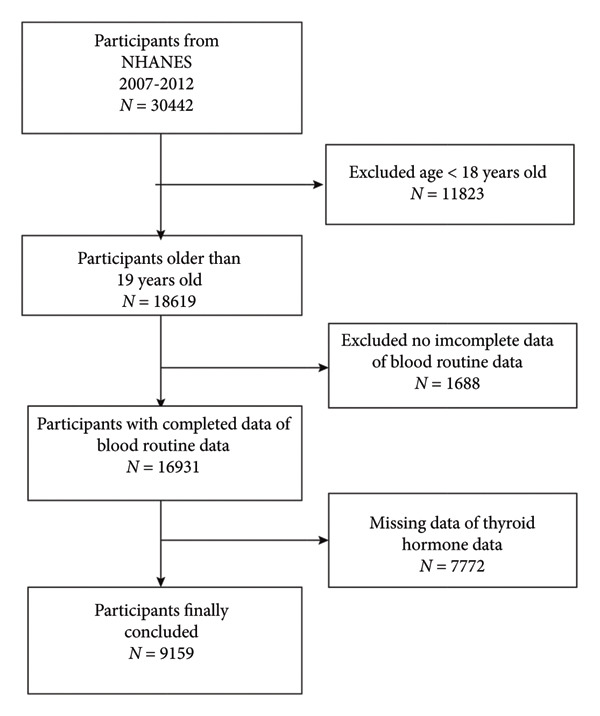
Flowchart of participant selection.

Among the 30,442 participants, we excluded 11,823 individuals aged < 18 years, 1688 without complete blood count data, and 7772 without TH measurements. Finally, 9159 participants were included in the final analysis (Figure 1).

### 2.2. Study Variables

#### 2.2.1. Assessment of THs

Serum THs were quantified with standardized immunoenzymatic assays. Total triiodothyronine (TT3), free triiodothyronine (FT3), and total thyroxine (TT4) were measured by competitive‐binding immunoenzymatic assays. Free thyroxine (FT4) was determined using a two‐step enzyme immunoassay. Thyroid‐stimulating hormone (TSH) concentrations were obtained with a two‐site (“sandwich”) immunoenzymatic assay. Thyroglobulin was assayed via a one‐step sandwich immunoenzymatic method, and thyroglobulin antibodies were assessed with a sequential two‐step sandwich immunoenzymatic assay.

#### 2.2.2. Definition of SII

Complete blood counts were obtained with an automated hematology analyzer (Coulter DxH 800 Analyzer). Platelet, neutrophil, and lymphocyte counts were determined by whole blood counts and presented as × 10^3^ cells/mL. The SII was then calculated as platelet count × neutrophil count/lymphocyte count, following the original description [22].

#### 2.2.3. Other Variables

This study assessed covariates that might have an influence on the relationship between SII and THs, including age, gender, race, education level, family income‐to‐poverty (PIR) ratio, smoking status, alcohol consumption, body mass index (BMI), waist circumference, hypertension (HBP) and diabetes history, serum albumin, blood urea nitrogen, creatinine, uric acid, alanine transaminase (ALT), aspartate aminotransferase (AST), glucose of serum, and iodine of urine.

### 2.3. Statistical Analyses

The statistical analysis was performed with R (version 4.2) and EmpowerStats (version 2.0) statistical computation. Descriptive statistics across SII quartiles were expressed as weighted means ± standard deviations for continuous variables and percentages for categorical variables; comparisons used weighted linear regression. Multivariate linear regression analysis calculated the *β*‐coefficients and 95% confidence intervals (CIs) between the SII and THs. Considering the potential impact of confounding factors on the study variables, two models were used to construct the multivariate test: Model 1: Age, gender, and race were adjusted; Model 2: Age, gender, race, PIR, alcohol, smoking, BMI, waist, HBP, diabetes history, red blood cell count, hemoglobin, glucose, albumin, blood urea nitrogen, creatinine, uric acid, ALT, AST, and iodine of urine were adjusted. Regression analysis demonstrated that SII was log‐transformed due to its right‐skewed distribution. Given the large sample size and data complexity, potential nonlinear relationships between lgSII and thyroid function parameters were explored. Smoothed curve fitting was performed using generalized additive models (GAMs), a nonparametric approach that does not assume a predefined functional form and allows for flexible data fitting. To quantify potential inflection points in these associations, threshold effect analysis was conducted using a two‐piecewise linear regression model. The threshold value for lgSII of FT3 and TSH was determined through a recursive algorithm that iteratively tested candidate breakpoints and selected the point that maximized the model log‐likelihood. The statistical significance of the threshold effect was evaluated using a log‐likelihood ratio test, comparing the two‐piecewise model with a simple linear model. This method is widely used in epidemiological studies to detect nonlinear associations in continuous variables. In addition, the gender subgroups were studied using the same statistical techniques previously mentioned. *P* < 0.05 was considered to be statistically significant.

## 3. Results

Table 1 presents weighted baseline characteristics across lgSII quartiles (Q1–Q4; *n* = 9159). Participants in the highest quartile (Q4) were older, more frequently non‐Hispanic White, and had lower PIR ratios. They also exhibited higher prevalence of hypertension and diabetes, along with elevated BMI, waist circumference, white blood cell count, neutrophils, and platelet counts. In contrast, lymphocyte counts, hemoglobin, FT3, TT3, and albumin concentrations were lower in Q4 (Table 1).

**Table 1:** Characteristics of the participants across lgSII quartiles.

Characteristics	Q1	Q2	Q3	Q4	*p-*Value
	*N* = 2290	*N* = 2289	*N* = 2290	*N* = 2290	
Age (years)	48.04 ± 18.81	47.73 ± 18.39	48.85 ± 18.12	49.45 ± 19.04	0.007^∗^
Gender (%)					< 0.001^∗^
Male	54.06%	50.42%	48.69%	45.46%	
Female	45.94%	49.58%	51.31%	54.54%	
Race (%)					< 0.001^∗^
Mexican American	14.19%	17.47%	18.65%	15.72%	
Other Hispanic	10.70%	11.62%	10.79%	11.18%	
Non‐Hispanic White	36.77%	45.04%	48.60%	51.79%	
Non‐Hispanic Black	30.66%	18.22%	15.76%	14.41%	
Other race	7.69%	7.65%	6.20%	6.90%	
Education (%)					0.167
Less than high school	13.20%	12.52%	12.38%	11.82%	
High school	39.01%	38.29%	39.57%	42.24%	
Above high school	47.79%	49.19%	48.06%	45.94%	
PIR	2.44 ± 1.63	2.59 ± 1.65	2.53 ± 1.63	2.34 ± 1.57	< 0.001^∗^
Body mass index (kg/m^2^)	28.08 ± 6.36	28.62 ± 6.35	28.92 ± 6.48	29.40 ± 7.61	< 0.001^∗^
Waist circumference (cm)	96.25 ± 15.73	97.76 ± 15.79	98.94 ± 15.80	100.12 ± 17.37	< 0.001^∗^
HBP (%)					< 0.001^∗^
Yes	32.66%	31.24%	34.45%	36.64%	
No	67.34%	68.76%	65.55%	63.36%	
Systolic blood pressure (mmHg)	124.33 ± 19.44	123.80 ± 18.73	124.23 ± 19.06	124.64 ± 18.79	0.548
Diastolic blood pressure (mmHg)	69.96 ± 13.11	70.30 ± 12.81	69.77 ± 13.24	69.46 ± 13.67	0.208
Diabetes (%)					0.045^∗^
Yes	11.79%	10.53%	11.75%	13.23%	
No	88.21%	89.47%	88.25%	86.77%	
Alcohol (%)					0.003^∗^
Yes	70.53%	71.95%	74.63%	69.71%	
No	29.47%	28.05%	25.37%	30.29%	
Smoking (%)					< 0.001^∗^
Yes	42.58%	44.78%	48.55%	49.91%	
No	57.42%	55.22%	51.45%	50.09%)	
White blood cell count (1000 cells/uL)	6.16 ± 2.64	6.74 ± 1.62	7.39 ± 1.85	8.49 ± 2.64	< 0.001^∗^
Lymphocyte (1000 cells/uL)	2.51 ± 1.98	2.51 ± 1.98	2.07 ± 0.64	1.82 ± 0.63	< 0.001^∗^
Segmented neutrophils (1000 cell/uL)	2.89 ± 0.95	3.75 ± 0.98	4.51 ± 1.20	5.84 ± 2.19	< 0.001^∗^
Red blood cell count (million cells/uL)	4.62 ± 0.51	4.68 ± 0.48	4.68 ± 0.50	4.63 ± 0.53	< 0.001^∗^
Hemoglobin (g/dL)	14.11 ± 1.46	14.25 ± 1.51	14.23 ± 1.55	14.00 ± 1.65	< 0.001^∗^
Platelet count (1000 cells/uL)	211.83 ± 52.71	242.70 ± 51.23	262.28 ± 54.74	298.28 ± 73.39	< 0.001^∗^
FT3 (pg/mL)	3.20 ± 0.77	3.20 ± 0.54	3.18 ± 0.47	3.13 ± 0.40	< 0.001^∗^
FT4 (ng/dL)	0.80 ± 0.19	0.80 ± 0.17	0.80 ± 0.16	0.81 ± 0.18	0.116
TT3 (ng/dL)	114.65 ± 26.25	114.25 ± 26.02	114.18 ± 26.78	112.00 ± 24.98	0.002^∗^
TT4 (ug/dL)	7.85 ± 1.75	7.89 ± 1.68	7.99 ± 1.54	8.15 ± 1.72	< 0.001^∗^
TSH (mIU/L)	2.12 ± 6.27	1.98 ± 2.59	2.02 ± 3.44	2.04 ± 3.00	0.696
Thyroglobulin antibodies (IU/mL)	10.47 ± 100.56	11.44 ± 97.48	10.23 ± 88.41	14.05 ± 115.56	0.564
Thyroglobulin (ng/mL)	16.30 ± 26.11	15.17 ± 24.16	16.53 ± 42.93	20.07 ± 107.64	0.039^∗^
Albumin (g/L)	42.80 ± 3.22	42.72 ± 3.14	42.54 ± 3.23	41.68 ± 3.88	< 0.001^∗^
Blood urea nitrogen (mg/dL)	13.06 ± 5.61	13.06 ± 5.35	13.08 ± 5.41	13.34 ± 6.69	0.285
Creatinine, serum (mg/dL)	0.91 ± 0.41	0.88 ± 0.32	0.89 ± 0.42	0.88 ± 0.38	0.032^∗^
Uric acid (mg/dL)	5.45 ± 1.42	5.50 ± 1.42	5.47 ± 1.42	5.53 ± 1.55	0.252
ALT (U/L)	26.54 ± 19.43	25.68 ± 16.16	25.93 ± 25.07	24.79 ± 26.49	0.061
AST (U/L)	27.99 ± 17.42	25.91 ± 12.62	25.75 ± 13.90	25.91 ± 26.36	< 0.001^∗^
Glucose, serum (mg/dL)	100.33 ± 36.19	99.58 ± 33.18	102.83 ± 41.26	104.55 ± 43.37	< 0.001^∗^
Iodine, urine (ug/L)	244.84 ± 1137.33	231.57 ± 646.81	232.73 ± 548.61	755.08 ± 16,464.15	0.087

*Note:* Mean ± SD for continuous variables: *p*‐Value was calculated by a weighted linear regression model; % for categorical variables: *p*‐Value was calculated by a weighted chi‐square test. *Q*, quartile; PIR, family income‐to‐poverty ratio; FT3, free triiodothyronine; FT4, free thyroxine; TT3, total triiodothyronine; TT4, total thyroxine; ALT, alanine transaminase; AST, aspartate aminotransferase.

Abbreviations: HBP, high blood pressure; TSH, thyroid‐stimulating hormone.

^∗^ *p* < 0.05.

Table 2 displays the results of the multiple linear regression analysis between thyroid function and lgSII. We found an inverse association between lgSII and FT3 (*β* = −0.13; 95% CI: −0.17 to −0.08), TT3 (*β* = −3.29; 95% CI: −5.46 to −1.13), and TSH (*β* = −0.60; 95% CI: −0.96 to −0.25) in Model 1 as shown in Table 2. This inverse association between lgSII and FT3 (*β* = −0.17, 95% CI: −0.22 to −0.11), TT3 (*β* = −3.06; 95% CI: −5.52 to −0.60), and TSH (*β* = −0.38; 95% CI: −0.71 to −0.06) remained significant even after adjusting for confounders in Model 2. However, this association between lgSII and TT4 became significantly positive in Model 1 (*β* = 0.47; 95% CI: 0.32–0.61) and Model 2 (*β* = 0.27, 95% CI: 0.10–0.44) as shown in Table 2. We also converted lgSII from a continuous variable into a categorical variable, as indicated by the sensitivity analysis result (lgSII quartiles, Q1–Q4). The trend testing between lgSII and FT3 (*p* for trend 0.0006) and TT3 (*p* for trend 0.0058) found a significant negative relationship in Model 1. In Model 2, the correlation between lgSII and FT3 (*p* for trend 0.0004) and TT3 (*p* for trend 0.0192) remained considerably negative as shown in Table 2. At the same time, lgSII had a highly positive correlation with the TT4 both in Model 1 (*p* for trend < 0.0001) and in Model 2 (*p* for trend 0.0008) (Table 2).

**Table 2:** Association between lgSII and thyroid hormone.

SII	FT3	FT4	TT3	TT4	TSH
Model 1 *β* (95%CI) *p* value					
lgSII	−0.13 (−0.17, −0.08) < 0.0001^∗^	0.01 (−0.00, 0.03) 0.0969	−3.29 (−5.46, −1.13) 0.0029^∗^	0.47 (0.32, 0.61) < 0.0001^∗^	−0.60 (−0.96, −0.25) 0.0009^∗^
Q1	Ref	Ref	Ref	Ref	Ref
Q2	−0.01 (−0.04, 0.02) 0.4229	−0.01 (−0.02, 0.00) 0.2306	−0.67 (−2.12, 0.77) 0.3607	0.04 (−0.05, 0.14) 0.3894	−0.22 (−0.46, 0.01) 0.0648
Q3	−0.01 (−0.05, 0.02) 0.3443	−0.00 (−0.02, 0.01) 0.3419	−0.30 (−1.75, 1.15) 0.6863	0.13 (0.03, 0.23) 0.0082^∗^	−0.22 (−0.46, 0.02) 0.0713
Q4	−0.05 (−0.08, −0.02) 0.0008^∗^	0.00 (−0.01, 0.01) 0.4526	−2.09 (−3.55, −0.64) 0.0049^∗^	0.29 (0.19, 0.39) < 0.0001^∗^	−0.22 (−0.46, 0.02) 0.0699
*p* for trend	0.0006^∗^	0.2430	0.0058^∗^	< 0.0001^∗^	0.1416

Model 2 *β* (95%CI) *p* value					
lgSII	−0.17 (−0.22, −0.11) < 0.0001^∗^	0.00 (−0.01, 0.02) 0.7418	−3.06 (−5.52, −0.60) 0.0149^∗^	0.27 (0.10, 0.44) 0.0016^∗^	−0.38 (−0.71, −0.06) 0.0204^∗^
Q1	Ref	Ref	Ref	Ref	Ref
Q2	−0.03 (−0.07, −0.00) 0.0493^∗^	−0.01 (−0.02, 0.00) 0.1626	−1.46 (−3.08, 0.16) 0.0773	0.02 (−0.09, 0.13) 0.7075	0.00 (−0.21, 0.21) 0.9920
Q3	−0.03 (−0.07, 0.00) 0.0746	−0.01 (−0.02, 0.00) 0.1009	−1.27 (−2.90, 0.35) 0.1241	0.04 (−0.07, 0.15) 0.4774	0.04 (−0.17, 0.25) 0.7151
Q4	−0.07 (−0.10, −0.03) 0.0002^∗^	−0.00 (−0.01, 0.01) 0.8002	−2.21 (−3.87, −0.55) 0.0090^∗^	0.18 (0.07, 0.30) 0.0017^∗^	0.00 (−0.21, 0.22) 0.9664
*p* for trend	0.0004^∗^	0.9441	0.0192^∗^	0.0008^∗^	0.9328

*Note:* Model 1: Adjusted for gender, age, and race. Model 2: Adjusted for age, gender, race, PIR, BMI, waist, HBP, diabetes, alcohol, smoking, red blood cell count, hemoglobin, glucose, albumin, blood urea nitrogen, creatinine, uric acid, ALT, AST, and iodine of urine; *Q*, quartile. FT3, free triiodothyronine; FT4, free thyroxine; TT3, total triiodothyronine; TT4, total thyroxine; lgSII, log‐transformed SII.

Abbreviations: CI, confidence interval; TSH, thyroid‐stimulating hormone.

^∗^ *p* < 0.05.

After subgroup analyses stratified by gender, the results indicate the inverse correlation between lgSII and FT3 (*β* = −0.28; 95% CI: −0.35 to −0.20) and the positive relationship between lgSII and TT4 (*β* = 0.29; 95% CI: 0.05–0.53) in Model 2 for females as shown in Table 3. However, it showed independent and significant positive relationship between lgSII and FT4 (*β* = 0.03; 95% CI: 0.00–0.05) and TT4 (*β* = 0.24; 95% CI: 0.01–0.47) and the negative relationship between lgSII and TT3 (*β* = −4.88; 95% CI: −8.32 to −1.45) and TSH (*β* = −0.86; 95% CI: −1.31 to −0.41) in Model 2 for males (Table 3).

**Table 3:** Subgroup analysis stratified by gender.

lg SII	FT3	FT4	TT3	TT4	TSH
Model 1 *β* (95%CI) *p* value					
Male	−0.03 (−0.09, 0.03) 0.3609	0.04 (0.02, 0.06) 0.0002^∗^	−5.26 (−8.31, −2.21) 0.0007^∗^	0.42 (0.22, 0.62) < 0.0001^∗^	−0.80 (−1.30, −0.29) 0.0019^∗^
Female	−0.21 (−0.28, −0.15) < 0.0001^∗^	−0.01 (−0.03, 0.01) 0.4034	−1.53 (−4.62, 1.56) 0.3309	0.52 (0.32, 0.72) < 0.0001^∗^	−0.44 (−0.95, 0.07) 0.0889

Model 2 *β* (95%CI) *p* value					
Male	−0.06 (−0.13, 0.02) 0.1324	0.03 (0.00, 0.05) 0.0337^∗^	−4.88 (−8.32, −1.45) 0.0053^∗^	0.24 (0.01, 0.47) 0.0453^∗^	−0.86 (−1.31, −0.41) 0.0002^∗^
Female	−0.28 (−0.35, −0.20) < 0.0001^∗^	−0.01 (−0.04, 0.01) 0.2549	−1.66 (−5.18, 1.86) 0.3552	0.29 (0.05, 0.53) 0.0188^∗^	0.04 (−0.42, 0.51) 0.8587

*Note:* Model 1: Adjusted for gender, age, and race. Model 2: Adjusted for age, gender, race, PIR, BMI, waist, HBP, diabetes, alcohol, smoking, red blood cell count, hemoglobin, glucose, albumin, blood urea nitrogen, creatinine, uric acid, ALT, AST, and iodine of urine. FT3, free triiodothyronine; FT4, free thyroxine; TT3, total triiodothyronine; TT4, total thyroxine; TSH, thyroid‐stimulating hormone; lgSII, log‐transformed SII.

Abbreviation: CI, confidence interval.

^∗^ *p* < 0.05.

Further utilization of the threshold effect analysis was used to explore the nonlinear connection between the TH and lgSII. The nonlinear relationship and saturation effect between the TH and lgSII were investigated by smooth curve fittings. When potential confounders were considered, an L‐shaped correlation was observed between FT3 and TSH and lgSII. The fitted graphs for the THs with lgSII exhibited inflection points. Using threshold effect analysis based on two‐piecewise linear regression, the inflection point (threshold) for lgSII was identified at 2.29 for FT3 and TSH (Figures 2(a), 2(e)). This result indicated that when lgSII < 2.29, lgSII was significantly negatively correlated with FT3 and TSH, whereas when lgSII > 2.29, the relationship between lgSII and FT3 and TSH showed a saturation effect and the correlation decreased. For TT4 (Figure 2(d)), lgSII was always positively correlated with it, and when lgSII > 2.71 as an inflection point, the correlation between them was significantly different. Similarly, FT4 and TT3 (Figures 2(b), 2(c)) showed significant correlations that appeared after the inflection point. The relevant results are detailed as shown in Table 4.

Figure 2The nonlinear relationship and saturation effect between the thyroid hormone and log‐transformed SII (lgSII). The red solid line represents the survey‐weighted smoothing spline estimate, and the blue dotted lines denote the 95% confidence interval. An L‐shaped correlation between FT3, TSH, and lgSII was observed (a and e). The inflection point of lgSII for the FT3 and TSH was finally found to be 2.29. Nonlinear associations between lgSII and FT4, TT3, and TT4, respectively (b, c, d).

**Figure:**
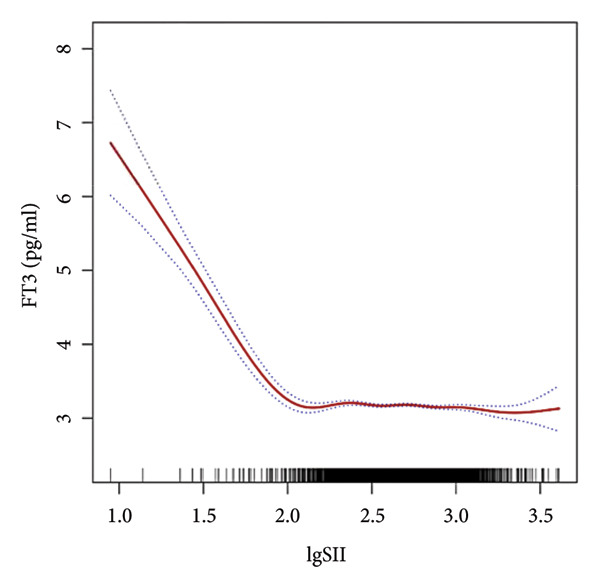
(a)

**Figure:**
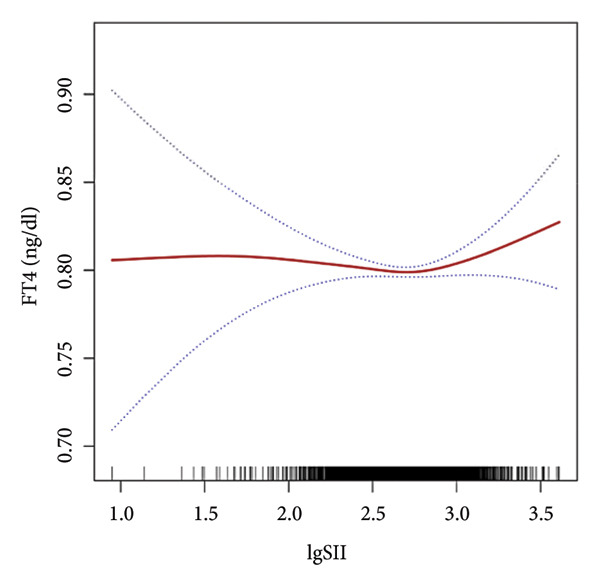
(b)

**Figure:**
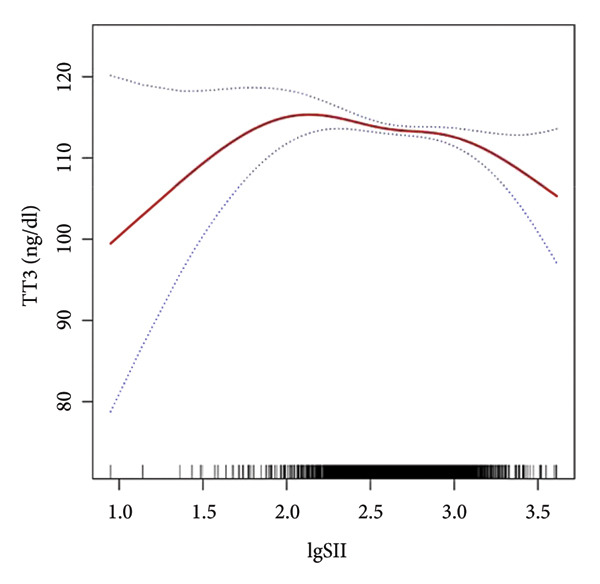
(c)

**Figure:**
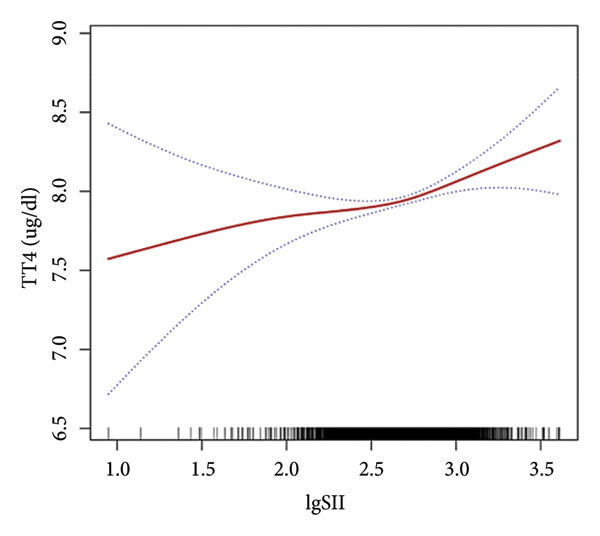
(d)

**Figure:**
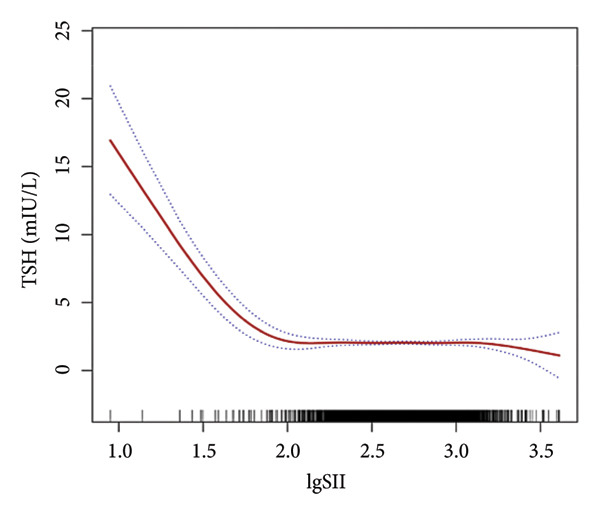
(e)

**Table 4:** Threshold effect analysis of lgSII and thyroid hormone using a two‐piecewise linear regression model.

lg SII	Adjusted β (95% CI) *p*‐value
*FT3*	
Inflection point	2.29
< Inflection point	−1.38 (−1.63, −1.14) < 0.0001
> Inflection point	−0.04 (−0.09, 0.02) 0.2205
Log‐likelihood ratio	< 0.001

*FT4*	
Inflection point	2.79
< Inflection point	−0.02 (−0.04, 0.01) 0.1523
> Inflection point	0.05 (0.01, 0.10) 0.0243
Log‐likelihood ratio	0.021

*TT3*	
Inflection point	3.05
< Inflection point	−2.11 (−4.81, 0.59) 0.1255
> Inflection point	−16.80 (−33.06, −0.53) 0.0430
Log‐likelihood ratio	0.093

*TT4*	
Inflection point	2.71
< Inflection point	0.04 (−0.24, 0.33) 0.7652
> Inflection point	0.58 (0.23, 0.93) 0.0011
Log‐likelihood ratio	0.046

*TSH*	
Inflection point	2.29
< Inflection point	−4.52 (−6.04, −2.99) < 0.0001
> Inflection point	0.06 (−0.30, 0.42) 0.7597
Log‐likelihood ratio	< 0.001

*Note:* FT3, free triiodothyronine; FT4, free thyroxine; TT3, total triiodothyronine; TT4, total thyroxine; lgSII, log‐transformed SII.

Abbreviations: CI, confidence interval; TSH, thyroid‐stimulating hormone.

## 4. Discussion

In this study, based on a cohort of U.S. adults from the NHANES 2007–2012 database, we observed that lgSII was inversely associated with FT3, TT3, and TSH, while showing a positive association with TT4. Moreover, we have demonstrated a distinctive L‐shaped relationship between lgSII and FT3 and TSH through the threshold effect analysis, in which the inflection point of lgSII was finally found to be 2.29.

Unlike conventional approaches, we explored the nonlinear correlation between lgSII and THs. This method has been increasingly applied in recent years. For example, Xia et al. found that the uric acid‐to‐high‐density lipoprotein cholesterol ratio was negatively correlated with total testosterone levels and positively associated with the risk of testosterone deficiency using smooth curve fits [23]. Similarly, Li et al. applied smooth curve analysis and found that plain water intake was negatively associated with periodontitis risk in middle‐aged and elderly adults in the United States [24]. This approach appears well‐suited for large cross‐sectional studies with potential confounding factors and has helped researchers to get numerous insightful findings, although it does not establish causal relationships between variables.

Our study indicates a potential association between SII and THs; however, the current studies regarding the relationship between SII and thyroid function remain limited. Similarly, Wang et al. conducted a study that revealed that individuals with thyroid dysfunction displayed increased levels of leukocytes, neutrophils, CRP, and calcitonin gene expression, alongside reduced lymphocyte counts [25]. Elevated inflammatory markers, which indicate systemic inflammation, have been associated with tissue damage, ultimately imparting deiodinase activity. Consequently, this decrease hinders the conversion of T4 to T3, leading to decreased FT3 levels [26]. Additionally, during sepsis, thyroidal T4 and T3 syntheses are downregulated by cytokines, with the severity of illness reflected by decreases in serum T3 [27]. Furthermore, studies have shown that in critical disease, chronic inflammation, and sepsis, the serum T3 levels may diminish to an exceedingly low point [28].

Our data revealed an inflection point at lgSII = 2.29, beyond which FT3 and TSH levels declined sharply and then plateaued, forming an L‐shaped pattern. This indicates that mild inflammatory activation exerts limited effects on thyroid function, whereas once systemic inflammation surpasses this threshold, both TH synthesis and peripheral T4‐to‐T3 conversion are markedly suppressed, consistent with early nonthyroidal illness syndrome (NTIS). THs serve as a crucial source of iodine and play an essential role in the antimicrobial activity of neutrophils through the myeloperoxidase–hydrogen peroxide–halide system. During the degradation of THs, the concentration of iodide increases within neutrophils, consequently enhancing the bactericidal effectiveness [29]. Beyond their antimicrobial functions, THs also regulate immune and inflammatory processes. Physiological levels of T3 can promote anti‐inflammatory responses and enhance macrophage bactericidal activity [30]. In contrast, abnormal T4 levels, such as in hypothyroidism, can activate proinflammatory pathways (e.g., PI3K–AKT), increase reactive oxygen species (ROS) production, and induce inflammatory responses [31]. These observations suggest that THs and inflammation interact through a bidirectional regulatory mechanism: Systemic inflammation suppresses TH activity, whereas altered TH signaling further modulates immune responses. As SII is a comprehensive indicator of systemic inflammation, it may reflect the inflammatory stress that disrupts TH homeostasis, providing a biological basis for the observed L‐shaped association. Recent reviews have emphasized this reciprocal relationship, demonstrating that THs act as active immunomodulators influencing cytokine production, macrophage polarization, and oxidative stress balance during systemic inflammation [18, 30, 32]. These findings provide a biological basis supporting the observed L‐shaped association between SII and TH homeostasis.

This study has several notable strengths. First, the inclusion of a large number of participants with an appropriate covariate correction significantly enhanced the study’s representativeness and reliability. Second, the application of sensitivity analyses effectively reduces the risk of false‐positive associations. Furthermore, our results identified an inflection point at lgSII = 2.29, beyond which the suppression of FT3 and TSH intensifies. Given that the SII can be easily calculated from routine complete blood counts and is cost‐effective, it may serve as a practical screening tool for identifying individuals with a high inflammatory burden who are at risk of subclinical thyroid dysfunction, thereby enabling earlier monitoring and targeted intervention.

Despite its strengths, this study has several limitations. First, as a cross‐sectional analysis, causal relationships between SII and THs cannot be inferred. Second, the NHANES cohort represents a specific population, which may limit the generalizability of our findings to other populations worldwide. Finally, the interplay between systemic inflammation and THs is complex and multifactorial. Future research should include mechanistic studies and well‐designed prospective cohort to further clarify these associations.

## 5. Conclusion

This study applied multiple statistical methods, including multivariable linear regression, smoothed curve fitting, and saturation effect analysis models, to investigate the relationship between SII and THs. Our findings revealed the nonlinear negative correlation between lgSII and FT3, TT3, and TSH, as well as a nonlinear positive correlation between lgSII and TT4. A saturation point was identified at lgSII = 2.29, indicating an L‐shaped relationship for FT3 and TSH. These results suggest that a higher inflammatory burden is associated with altered TH homeostasis. Clinically, given that SII can be easily calculated from a routine complete blood count, it may serve as a cost‐effective and easily accessible screening tool to identify individuals at risk of subclinical thyroid dysfunction, enabling earlier monitoring and potential targeted intervention. Future prospective studies or randomized controlled trials are warranted to further investigate the causal relationship between SII and thyroid function.

## Disclosure

All authors approved the final version for publication.

## Conflicts of Interest

The authors declare no conflicts of interest.

## Author Contributions

Jiaqi Huang contributed significantly to the conception and drafting of the work, Jieqiong Song was responsible for data acquisition and drafting, Ming Zhong conducted data analysis and critical revision, and Fei Leng performed data interpretation and critical review. All authors take responsibility for the work’s integrity. Jiaqi Huang and Jieqiong Song contributed equally to this study.

## Funding

This work was supported by the Excellent Training Program of Minhang Hospital, the Fudan University (2023MHPY03), and the Youth Funding of Zhongshan Hospital Fudan University (2024ZSQN31).

## Data Availability

The data that support the findings of this study are publicly available from the National Health and Nutrition Examination Survey (NHANES) at https://wwwn.cdc.gov/nchs/nhanes/.

## References

[1] Hu B. , Yang X.-R. , Xu Y. et al., Systemic Immune-Inflammation Index Predicts Prognosis of Patients After Curative Resection for Hepatocellular Carcinoma, Clinical Cancer Research. (2014) 20, no. 23, 6212–6222, 10.1158/1078-0432.CCR-14-0442, 2-s2.0-84918495553. 25271081

[2] Chen J.-H. , Zhai E.-T. , Yuan Y.-J. et al., Systemic Immune-Inflammation Index for Predicting Prognosis of Colorectal Cancer, WJG. (2017) 23, no. 34, 10.3748/wjg.v23.i34.6261, 2-s2.0-85029591266. PMC5603492 28974892

[3] Huang H. , Liu Q. , Zhu L. et al., Prognostic Value of Preoperative Systemic Immune-Inflammation Index in Patients with Cervical Cancer, Scientific Reports. (2019) 9, no. 1, 10.1038/s41598-019-39150-0, 2-s2.0-85062348899. PMC6397230 30824727

[4] He L. , Xie X. , Xue J. , Xie H. , and Zhang Y. , Association of the Systemic Immune-Inflammation Index with All-Cause Mortality in Patients with Arteriosclerotic Cardiovascular Disease, Frontiers in Cardiovascular Medicine. (2022) 9, 10.3389/fcvm.2022.952953. PMC9510918 36172591

[5] Tian B.-W. , Yang Y.-F. , Yang C.-C. et al., Systemic Immune-Inflammation Index Predicts Prognosis of Cancer Immunotherapy: Systemic Review and Meta-Analysis, Immunotherapy. (2022) 14, no. 18, 1481–1496, 10.2217/imt-2022-0133. 36537255

[6] Yang Y. , Wu C. , Hsu P. et al., Systemic Immune‐Inflammation Index (SII) Predicted Clinical Outcome in Patients with Coronary Artery Disease, European Journal of Clinical Investigation. (2020) 50, no. 5, 10.1111/eci.13230. 32291748

[7] Mahemuti N. , Jing X. , Zhang N. et al., Association Between Systemic Immunity-Inflammation Index and Hyperlipidemia: a Population-based Study from the NHANES (2015–2020), Nutrients. (2023) 15, no. 5, 10.3390/nu15051177. PMC10004774 36904176

[8] Benz E. , Wijnant S. R. A. , Trajanoska K. et al., Systemic Immune-Inflammation Index and All-Cause Mortality in Middle-Aged and Older People with COPD and Asthma: a Population-Based Study, ERJ Open Res. (2022) 8, no. 1, 00628–02021, 10.1183/23120541.00628-2021. 35036418 PMC8752940

[9] Lai W. , Xie Y. , Zhao X. et al., Elevated Systemic Immune Inflammation Level Increases the Risk of Total and Cause-Specific Mortality Among Patients with Chronic Kidney Disease: a Large Multi-Center Longitudinal Study, Inflammation Research. (2023) 72, no. 1, 149–158, 10.1007/s00011-022-01659-y. 36352033

[10] Di X. , Liu S. , Xiang L. , and Jin X. , Association Between the Systemic Immune-Inflammation Index and Kidney Stone: a Cross-Sectional Study of NHANES 2007-2018, Frontiers in Immunology. (2023) 14, 10.3389/fimmu.2023.1116224. PMC9989007 36895572

[11] Okutan İ. , Aci R. , Keskin Â. , Bilgin M. , and Kızılet H. , New Inflammatory Markers Associated with Disease Activity in Rheumatoid Arthritis: Pan-Immune-Inflammation Value, Systemic immune-inflammation Index, and Systemic Inflammation Response Index, Reumatologia. (2024) 62, no. 6, 439–446, 10.5114/reum/196066. 39866302 PMC11758101

[12] Zhang Y. , Meng Y. , Chen M. et al., Correlation Between the Systemic Immune-Inflammation Indicator (SII) and Serum Ferritin in US Adults: a Cross-Sectional Study Based on NHANES 2015-2018, Annals of Medicine. (2023) 55, no. 2, 10.1080/07853890.2023.2275148. PMC10836291 37883981

[13] Aci R. , Keskin A. , Türe E. , and Türe E. , Inflammatory Markers and Gestational Diabetes Mellitus Risk: Investigating neutrophil/lymphocyte Ratio, platelet/lymphocyte Ratio, and Systemic Immune Inflammation Index, Ain Shams Medical Journal. (2024) 75, no. 3, 646–651, 10.21608/asmj.2024.290292.1273.

[14] Senese R. , Cioffi F. , Petito G. , Goglia F. , and Lanni A. , Thyroid Hormone Metabolites and Analogues, Endocrine. (2019) 66, no. 1, 105–114, 10.1007/s12020-019-02025-5, 2-s2.0-85069970852. 31359245

[15] Delitala A. P. , Fanciulli G. , Maioli M. , and Delitala G. , Subclinical Hypothyroidism, Lipid Metabolism and Cardiovascular Disease, European Journal of Internal Medicine. (2017) 38, 17–24, 10.1016/j.ejim.2016.12.015, 2-s2.0-85009273483. 28040402

[16] Lee K. , Lim S. , Park H. et al., Subclinical Thyroid Dysfunction, Bone Mineral Density, and Osteoporosis in a Middle-Aged Korean Population, Osteoporosis International. (2020) 31, no. 3, 547–555, 10.1007/s00198-019-05205-1. 31720711

[17] van Vliet N. A. , van Heemst D. , Almeida O. P. et al., Thyroid Studies Collaboration. Association of Thyroid Dysfunction with Cognitive Function: an Individual Participant Data Analysis, JAMA Internal Medicine. (2021) 181, no. 11, 1440–1450, 10.1001/jamainternmed.2021.5078. 34491268 PMC8424529

[18] Mancini A. , Di Segni C. , Raimondo S. et al., Thyroid Hormones, Oxidative Stress, and Inflammation, Mediators of Inflammation. (2016) 2016, 1–12, 10.1155/2016/6757154, 2-s2.0-84962432715. PMC4802023 27051079

[19] Vural S. , Muhtaroğlu A. , and Güngör M. , Systemic immune-inflammation Index: a New Marker in Differentiation of Different Thyroid Diseases, Medicine (Baltimore). (2023) 102, no. 31, 10.1097/MD.0000000000034596. PMC10402992 37543770

[20] Sorrenti S. , Scerrino G. , Lori E. et al., Inflammation and Thyroid Cancer: Deciphering the Role of Blood Immune Indexes, Cancers (Basel). (2025) 17, no. 8, 10.3390/cancers17081363. PMC12025745 40282539

[21] Çiftel S. and Tüzün Z. , Could the Systemic Immune Inflammation Index Predict Diagnosis, Recovery Time, Hypothyroidism, and Recurrence Rates in Subacute Thyroiditis?, International Journal of General Medicine. (2023) 16, 1375–1382, 10.2147/IJGM.S406724. 37096201 PMC10122476

[22] Xie Y. , Zhuang T. , Ping Y. et al., Elevated Systemic Immune Inflammation Index Level Is Associated with Disease Activity in Ulcerative Colitis Patients, Clinica Chimica Acta. (2021) 517, 122–126, 10.1016/j.cca.2021.02.016. 33662359

[23] Xia W. , Xie P. , Zhuang Q. et al., Association Between Uric acid/high-density Lipoprotein Cholesterol Ratio and Testosterone Deficiency in Adult American Men: Findings from the National Health and Nutrition Examination Survey 2011-2016, BMC Public Health. (2025) 25, no. 1, 10.1186/s12889-025-22194-5. PMC11892185 40065268

[24] Li X. , Wang L. , Yang L. , Liu X. , Liu H. , and Mu Y. , The Association Between Plain Water Intake and Periodontitis in the Population Aged over 45: a cross-sectional Study Based on NHANES 2009-2014, BMC Oral Health. (2024) 24, no. 1, 10.1186/s12903-023-03809-y. PMC10770954 38183113

[25] Wang W. , Su X. , Ding Y. et al., Thyroid Function Abnormalities in COVID-19 Patients, Frontiers in Endocrinology. (2021) 11, 10.3389/fendo.2020.623792. PMC7933556 33679608

[26] Ilera V. , Delfino L. C. , Zunino A. et al., Correlation Between Inflammatory Parameters and Pituitary–Thyroid Axis in Patients with COVID-19, Endocrine. (2021) 74, no. 3, 455–460, 10.1007/s12020-021-02863-2. 34515958 PMC8436010

[27] Bartalena L. , Bogazzi F. , Brogioni S. , Grasso L. , and Martino E. , Role of Cytokines in the Pathogenesis of the Euthyroid Sick Syndrome, European Journal of Endocrinology. (1998) 138, no. 6, 603–614, 10.1530/eje.0.1380603, 2-s2.0-0031863759. 9678522

[28] Boelen A. , Kwakkel J. , and Fliers E. , Beyond Low Plasma T3: Local Thyroid Hormone Metabolism During Inflammation and Infection, Endocrine Reviews. (2011) 32, no. 5, 670–693, 10.1210/er.2011-0007, 2-s2.0-83355164457. 21791567

[29] Slag M. F. , Hypothyroxinemia in Critically III Patients as a Predictor of High Mortality, JAMA. (1981) 245, no. 1, 10.1001/jama.1981.03310260021020, 2-s2.0-84948722591. 7431627

[30] Lasa M. and Contreras-Jurado C. , Thyroid Hormones Act as Modulators of Inflammation Through Their Nuclear Receptors, Frontiers in Endocrinology. (2022) 13, 10.3389/fendo.2022.937099. PMC9393327 36004343

[31] De Luca R. , Davis P. J. , Lin H.-Y. et al., Thyroid Hormones Interaction with Immune Response, Inflammation and Non-Thyroidal Illness Syndrome, Frontiers in Cell and Developmental Biology. (2021) 8, 10.3389/fcell.2020.614030. PMC7859329 33553149

[32] Wenzek C. , Boelen A. , Westendorf A. M. , Engel D. R. , Moeller L. C. , and Führer D. , The Interplay of Thyroid Hormones and the Immune System-where We Stand and Why We Need to Know About It, European Journal of Endocrinology. (2022) 186, no. 5, R65–R77, 10.1530/EJE-21-1171. 35175936 PMC9010816

